# Effect of Betahistine and Metformin on Antipsychotic-Induced Weight Gain: An Analysis of Two Clinical Trials

**DOI:** 10.3389/fpsyt.2018.00620

**Published:** 2018-11-27

**Authors:** Dongyu Kang, Zhihui Jing, Ranran Li, Gangrui Hei, Tiannan Shao, Li Li, Mengxi Sun, Ye Yang, Ying Wang, Xiaoyi Wang, Yujun Long, Xiansheng Huang, Renrong Wu

**Affiliations:** ^1^Department of Psychiatry, The Second Xiangya Hospital, Central South University, Changsha, China; ^2^Mental Health Institute of the Second Xiangya Hospital, Central South University, Changsha, China; ^3^The China National Clinical Research Center for Mental Health Disorders, Changsha, China; ^4^National Technology Institute of Psychiatry, Changsha, China; ^5^Key Laboratory of Psychiatry and Mental Health of Hunan Province, Changsha, China; ^6^Department of Cardiovascular Medicine, The Second Xiangya Hospital, Central South University, Changsha, China; ^7^Shanghai Institute for Biological Science, Chinese Academy of Sciences, Shanghai, China

**Keywords:** betahistine, metformin, BMI, insulin resistance, antipsychotic medication

## Abstract

Antipsychotic-induced weight gain is one of the most common adverse effects of antipsychotic treatment. However, there are no well-established interventions for the weight gain yet. In this study, we pooled the data from two clinical trials, which were originally examining the efficacy of betahistine and the efficacy of metformin in treating antipsychotic-induced weight gain and insulin resistance. A total of 67 people with schizophrenia or bipolar disorder treated with antipsychotics were assigned to 36 mg day^−1^ betahistine (*n* = 13) or 1,000 mg day^−1^ metformin (*n* = 25) or placebo (*n* = 29) treatment for 12 weeks, with evaluation at baseline and week 12. The primary outcome was the body mass index (BMI). After treatment, metformin group had a mean decrease in BMI of 1.46 ± 0.14 (*p* < 0.001) and insulin resistance index (IRI) of 4.30 ± 2.02 (*p* < 0.001). The betahistine group had no significant alteration in BMI or IRI. However, placebo group had a mean increase in BMI of 1.27 ± 0.77 (*p* < 0.001) and IRI of 0.45 ± 0.86 (*p* < 0.001). Between the two treatment groups, metformin significantly decreased weight, BMI, fasting glucose, insulin level, and IRI but not waist circumference when compared with betahistine. Moreover, metformin significantly decreased weight, BMI, waist circumference, fasting glucose, insulin level, and IRI when compared with placebo, whereas betahistine significantly decreased body weight, waist circumference, BMI, insulin level, and IRI but not fasting glucose when compared with placebo. In this study, we found that both metformin treatment and betahistine treatment were efficacious in improving antipsychotic-induced weight gain and insulin resistance, and metformin was more efficacious in preventing and revising the weight gain induced by antipsychotics.

**Clinical Trial Registration:** www.ClinicalTrials.gov, NCT00451399(Study 1), NCT00709202(Study 2)

## Introduction

Antipsychotics, especially second-generation antipsychotics (1), could lead to serious metabolic adverse effects, including weight gain, insulin resistance, and glucose intolerance (2, 3). These adverse effects significantly increase the risk of stroke and coronary artery disease (4) and make patients 13 times more likely to discontinue medication (5). Interestingly, previous studies found that overall 23.5% of the patients with schizophrenia had metabolic syndrome (6), and 78.8% of the patients receiving antipsychotic medication showed more than 7% increase in body weight compared with baseline (7). In addition to schizophrenia, bipolar disorder is also commonly treated with antipsychotics, and individuals with bipolar disorder and schizophrenia have similar chances of showing weight gain and metabolic syndrome (8). Meanwhile, one study suggested that people with severe mental illness may not necessarily have a higher risk for cardiovascular diseases while other factors like dietary habits, health coverage, and family's support are possible explanations for higher risk (9). Therefore, antipsychotic-induced weight gain has been a major management problem for clinicians when treating schizophrenia and bipolar disorder (10).

Metformin, a biguanide, is commonly used for type II diabetes mellitus. It functions by inhibiting hepatic gluconeogenesis and improving the sensitivity of insulin in skeletal muscles (11). Previous work in our lab indicated that metformin can decrease antipsychotic-induced weight gain and insulin resistance (12–15), potentially by reducing insulin resistance (16) and suppressing appetite (17). Betahistine, an antagonist of histaminergic H_1_ and H_3_ receptors, has been widely used to treat vertigo symptoms in Meniere's disease. Interestingly, a previous study suggested that histaminergic H1 and H3 receptors are crucial to the potential mechanism of antipsychotic-induced weight gain (18). Additionally, previous studies found that the coadministration of olanzapine and betahistine in rats significantly reduced olanzapine-induced weight gain (19, 20), and clinical trials showed that the coadministration of olanzapine and betahistine significantly reduced weight gain in individuals with schizophrenia or schizoaffective disorder (21, 22).

Despite overwhelming evidence on the effects of metformin and betahistine in treating weight gain associated with many disorders, it remains unknown which medication is more efficacious in treating antipsychotic-induced weight gain and insulin resistance in people with schizophrenia or bipolar disorder. Therefore, we analyzed data from two studies to compare the efficacies of metformin and betahistine on improving antipsychotic-induced weight gain and insulin resistance in people with schizophrenia or bipolar disorder. We hypothesized that both betahistine and metformin could attenuate antipsychotic-induced weight gain and insulin resistance in these patients.

## Materials and methods

### Study design

The study was designed using an independent double-blind, randomized, placebo-controlled 12-week clinical trial of metformin and an open label prospective cohort study of betahistine in weight gain and other metabolic changes. The first trial (NCT00451399) was to examine the efficacy of metformin in the treatment of antipsychotic-induced weight gain; the second trial (NCT00709202) originally was to examine the efficacy of betahistine in the treatment of antipsychotic-induced weight gain. The data from STUDY 2 have not been published elsewhere, though the primary outcomes of STUDY 1 have been published before (12).

The two studies were identical in terms of measurements of body weight, fasting glucose level, and other serum chemicals. In both STUDY1 and STUDY 2, participants had to gain more than 10% of their pre-drug body weight within the first year of treatment to be eligible for our study. Both studies were conducted in the Mental Health Institution of the Second Xiangya Hospital at the Central South University, China. In STUDY 1, 32 participants were randomly assigned to each group. After 12 weeks, 30 participants in metformin group completed the treatment, and 29 participants in placebo group completed the follow-up. In STUDY 2, there were 31 participants involved and 18 of them completed the treatment. After excluding 5 patients from metformin group who were not compliant and 5 patients from STUDY 2 whose course of illness was beyond 10 years, totally 54 patients from STUDY 1 and 13 patients from STUDY 2 were included for data analysis.

### Participants

People aged 18 through 55 diagnosed with schizophrenia or bipolar disorder in accordance with the criteria set out in the Diagnostic and Statistical Manual of Mental Disorders-Fourth Edition (DSM-IV) were eligible for our study (23), and the Structured Clinical Interview of DSM-IV Axis I Disorders (SCID-1), clinical version (24), was used during the screening phase. All the participants were on a stable dose of antipsychotics for at least 3 months before the start of the study, and it remained at the same dose throughout the course of the study. To be qualified for STUDY 2, the participants had to meet the following criteria: (1) the participants should have gained 10% of their body weight within the first year of treatment or within the last year if the treatment is beyond a year; and (2) the participants should have taken one of the four antipsychotics—clozapine, olanzapine, risperidone, or quetiapine. All the participants had to be taken care of by a parent or another adult caregiver who monitored and recorded the intake of medication daily. All the participants were recruited from the outpatient clinic of the Mental Health Institute of the Second Xiangya Hospital, Central South University, China, between March 2015 and March 2017. Participants were excluded from the study if there was evidence of liver or kidney dysfunction, asthma, peptic ulcer, or pheochromocytoma; if they were taking prescription medication, which affects body weight or glycolipid metabolism; if they were taking thyroid replacement therapy or lipid-regulatory drugs, whose dose changed more than 50% over the last 3 months; or if they were pregnant or lactating. For STUDY 1, the inclusion and exclusion criteria have been previously described (12). For both studies, after a complete description of the study to the subjects, written informed consent was obtained in accordance with the guidelines of the National Health and Medical Research Council. The study was approved by the Ethics Committee of the Second Xiangya Hospital.

### Pharmacological intervention

For STUDY 1, the participants were administered with the drug in a double-blind placebo controlled fashion. For the first 4 days, the participants took 250 mg of metformin or placebo at their evening meal, after which a second or third dose was added before breakfast and lunch, respectively, for another 80 days. Each participant was given a record sheet to log the number of trial medications taken daily. Only trihexyphenidyl for extrapyramidal symptoms or lorazepam for insomnia or agitation were allowed as needed. For STUDY 2, the participants took 6 mg of betahistine at their evening meal for the first 2 days after enrollment, then 6 mg of betahistine at lunch and evening meal for the third day, after which 6 mg more of betahistine was added each day till the 11th day. The participants took 18 mg of betahistine at lunch and evening meal at 11th day and for another 73 days. Any antipsychotics taken by the participants before enrollment remained at the same dosage throughout the course of the study.

### Primary and secondary outcome measurements

The primary outcome was body mass index (BMI). The secondary outcome was body weight, waist circumference, fasting glucose, insulin level, and insulin resistance index (IRI). To calculate the BMI, weight in kilograms was divided by height in meters squared. To calculate the IRI, insulin level (mIU/L) × fasting glucose (mmol/L)/22.5 was determined, in accordance with homeostasis model assessment (25).

Baseline data included related demographics, a comprehensive medical history, a physical examination with the measurement of weight and height, and a related laboratory test. Research nurses were blind to the type of treatment to perform all assessments. Related laboratory tests included fasting insulin and fasting glucose. Fasting blood samples were confirmed by the patients or their caregivers.

Follow-up visits were made at week 12 after starting the treatment, and all baseline evaluations including physical examination, laboratory test, and weight and height measurements were repeated. Serum insulin level was measured with a solid phase radioimmunoassay. Weight and height measurements were taken after removing their shoes and upper garments and donning an examination gown.

### Statistical analysis

All analyses were conducted by using the Statistical Package for Social Sciences, version 23 (SPSS Inc, Chicago, Illinois). Continuous variables were described as mean (SD) and confidence interval. Categorical variables were described by frequencies and percentages. We used *t*-tests, chi-squared analyses, and one-way analysis of variance (ANOVA) as appropriate. Fisher's exact test was used when necessary. For the comparison of the three groups at baseline, ANOVA was used to compare continuous variables with homogeneity of variance, Kruskal-Wallis test was conducted for the variables without homogeneity of variance, and chi-squared analysis was used for categorical variables.

For the follow-up data were all continuous variables, analysis of covariance (ANCOVA) was the main strategy used when we compared among the three groups, while the corresponding baseline values and those variables found to be significantly different at baseline were regarded as covariates. To compare the difference among the three groups, *post-hoc* tests (least significant difference procedure) were conducted to compare between the two groups. For the variables without homogeneity of variance, the ANOVA was conducted after ranking. The difference was considered statistically significant if a two-tailed *P*-value was < 0.05. The difference between baseline data and 12 weeks data was analyzed by a paired *t*-test.

## Results

### Demographic and baseline outcome measurements

Demographic and baseline outcomes were compared among the three groups (Table 1). The 25 patients (13 females and 12 males) of metformin group and 29 patients (15 females and 14 males) of placebo group were from STUDY 1, while the 13 patients (10 females and 3 males) of betahistine group were from STUDY 2. There were both schizophrenia and bipolar disorder participants in betahistine group, while only schizophrenia participants in metformin group and placebo group. No significant difference was found in the mean values of age, gender, medication, and IRI, while betahistine group had significantly higher values of illness duration, body weight, BMI, waist circumstance, fasting glucose, and insulin level than the other groups. Therefore, those seven outcomes in baseline were set as covariances in the data analysis. Betahistine group had a significantly longer course of illness than other two groups; therefore, we set the course of illness as the covariance in the data analysis. However, after ANCOVA, the course of illness was not statistically significant in any of the outcomes as a covariance.

**Table 1:** Demographic and clinical characteristics of 67 Participants across treatment groups at baseline^a^.

**Characteristics**	**Total (*n* = 67)**	**Betahistine (*n* = 13)**	**Metformin (*n* = 25)**	**Placebo (*n* = 29)**	**Test statistics^b^**	***P*-value**
Age	26.03 ± 5.11	26.23 ± 7.41	26.78 ± 4.30	25.84 ± 4.80	0.053	0.950
Gender(Male/Female)	(29/38)	(3/10)	(12/13)	(14/15)	2.683	0.260
Diagnose (schizophrenia/Bipolar disorder)	(59/8)	(5/8)	(25/0)	(29/0)	–	< 0.001
Medication	(19/17/20/11)	(0/6/5/2)	(9/5/7/4)	(10/6/8/5)	–	0.177
Clozapine	19	0 (3.7)	9 (7.1)	10 (8.2)	
Olanzapine	17	6 (3.3)	5 (6.3)	6 (7.4)	
Risperidone	20	5 (3.9)	7 (7.5)	8 (8.7)	
Quetiapine	11	2 (2.1)	4 (4.1)	5 (4.8)	
Course, Mo	14.31 ± 15.41	35.54 ± 25.98	9.28 ± 2.51	9.14 ± 2.36	19.738	< 0.001
Body weight	66.93 ± 9.04	76.25 ± 13.88	65.62 ± 5.78	63.88 ± 5.46	11.652	0.003
BMI	25.23 ± 2.33	28.38 ± 3.09	24.90 ± 1.10	24.09 ± 1.25	21.478	< 0.001
Waist circumference	86.76 ± 8.52	99.47 ± 8.20	84.22 ± 5.78	83.25 ± 4.50	38.473	0.001
Fasting Glucose	5.22 ± 0.49	4.91 ± 0.52	5.44 ± 0.47	5.17 ± 0.41	5.961	0.004
Insulin	26.64 ± 11.48	26.42 ± 21.76	28.21 ± 8.08	25.39 ± 6.93	6.282	0.043
IRI	6.28 ± 3.03	5.88 ± 5.31	6.96 ± 2.56	5.87 ± 1.81	4.075	0.130

BMI, body mass index, which is calculated as weight in kilograms divided by height in meters squared; IRI, insulin resistance index, which is calculated as insulin level(mIU/L) × fasting glucose(mmol/L)/22.5.

SI conversions: To convert glucose from mg/dL to mmol/L, multiply by 0.0555; insulin from μIU/mL to pmol/L, multiply by a 6.945.

a Data are presented as mean(SD).

b *Test statistics: χ^2^ Test and Fisher's exact test for categorical variables and analysis of variance for continuous variables*.

### Changes in body weight and body mass index

After 12 weeks of treatment, statistical analysis showed that, in metformin group, the mean body weight and BMI value decreased significantly, while in placebo group, there's a significant increase of body weight and BMI; however, in betahistine group, no significant differences were found in the mean body weight or BMI value (Table 2). After 12 weeks of treatment, metformin was significantly superior to betahistine and placebo in terms of values of body weight and BMI, while betahistine was superior to placebo in terms of values of body weight and BMI (Table 3).

**Table 2:** Treatment outcomes for all 67 participants.

	**Baseline**	**Endpoint**	***P*-Value**
**Betahistine group (*****n*** = **13)**			
Body weight, kg	76.25 ± 13.88 (67.86 – 84.643)	75.25 ± 12.90 (67.46 – 83.05)	0.223
BMI	28.38 ± 3.09 (26.51 – 30.25)	28.05 ± 3.19 (26.13 – 29.98)	0.245
Waist Circumference, cm	99.47 ± 8.20 (94.52 – 104.42)	98.65 ± 9.63 (92.83 – 104.46)	0.495
Fasting Glucose, mmol/L	4.91 ± 0.52 (4.60 – 5.23)	4.90 ± 0.61 (4.53 – 5.27)	0.920
Insulin, mIU/L	26.42 ± 21.76 (13.27 – 39.56)	20.34 ± 14.61 (11.51 – 29.17)	0.111
IRI	5.88 ± 5.31 (2.67 – 9.09)	4.48 ± 3.29 (2.50 – 6.47)	0.132
**Metformin group (*****n*** = **25)**			
Body weight, kg	65.62 ± 5.78 (63.25 – 68.01)	61.82 ± 6.26 (59.23 – 64.40)	< 0.001
BMI	24.90 ± 1.10 (24.45 – 25.36)	23.44 ± 1.31 (22.90 – 23.98)	< 0.001
Waist Circumference, cm	84.22 ± 5.78 (81.83 – 86.60)	82.69 ± 5.80 (80.30 – 85.09)	< 0.001
Fasting Glucose, mmol/L	5.44 ± 0.47 (5.24 – 5.63)	4.63 ± 0.65 (4.36 – 4.90)	< 0.001
Insulin, mIU/L	28.21 ± 8.08 (24.87 – 31.55)	12.87 ± 3.90 (11.26 – 14.47)	< 0.001
IRI	6.96 ± 2.56 (5.90 – 8.02)	2.66 ± 0.89 (2.29 – 3.02)	< 0.001
**Placebo group (*****n*** = **29)**			
Body weight, kg	63.88 ± 5.46 (61.80 – 65.96)	67.17 ± 5.28 (65.16 – 69.18)	< 0.001
BMI	24.09 ± 1.25 (23.61 – 24.56)	25.35 ± 1.16 (24.91 – 25.80)	< 0.001
Waist Circumference, cm	83.25 ± 4.50 (81.54 – 84.97)	85.54 ± 5.43 (83.47 – 87.60)	< 0.001
Fasting Glucose, mmol/L	5.17 ± 0.41 (5.01 – 5.33)	5.11 ± 0.40 (4.96 – 5.27)	0.081
Insulin, mIU/L	25.39 ± 6.93 (22.76 – 28.03)	27.62 ± 7.33 (24.83 – 30.40)	0.001
IRI	5.87 ± 1.81 (5.18 – 6.55)	6.32 ± 2.00 (5.56 – 7.08)	0.008

BMI, body mass index, which is calculated as weight in kilograms divided by height in meters squared; IRI, insulin resistance index, which is calculated as insulin level (mIU/L) × fasting glucose (mmol/L)/22.5.

*SI conversions: To convert glucose from mg/dL to mmol/L, multiply by 0.0555; insulin from μIU/mL to pmol/L, multiply by a 6.945*.

**Table 3:** The difference between baseline and end point of all treatment outcomes.

**Mean(SD) CI**				***P*****-value**				
**Assessment levels**	**Betahistine (*n* = 13)**	**Metformin (*n* = 25)**	**Placebo (*n* = 29)**	**ANCOVA^a^**	**Partial η^2^**	**Betahistine vs. Metformin**	**Betahistine vs. Placebo**	**Metformin vs. Placebo**
Body weight, kg	−1.00 ± 2.81 (−2.70, 0.70)	−3.80 ± 1.71 (−4.51, −3.10)	3.29 ± 1.92 (2.56, 4.02)	< 0.001	0.752	0.002	0.001	< 0.001
Waist circumference, cm	−0.82 ± 4.21 (−3.37, 1.72)	−1.52 ± 0.07 (−1.55, −1.50)	2.29 ± 1.58 (1.68, 2.89)	< 0.001	0.628	0.058	< 0.001	< 0.001
BMI	−0.33 ± 0.96 (−0.91, 0.26)	−1.46 ± 0.70 (−1.75, −1.17)	1.27 ± 0.77 (0.97, 1.56)	< 0.001	0.743	< 0.001	0.009	< 0.001
Fasting glucose, mmol/L	−0.013 ± 0.46 (−0.29, 0.26)	−0.81 ± 0.81 (−1.14, −0.48)	−0.05 ± 0.16 (−0.11, 0.01)	< 0.001	0.575	< 0.001	0.942	< 0.001
Insulin, mIU/L	−8.00 ± 14.93 (−17.02, 1.03)	−15.34 ± 5.57 (−17.64, −13.05)	2.22 ± 3.12 (1.04, 3.41)	< 0.001	0.741	0.002	< 0.001	< 0.001
IRI	−1.40 ± 3.12 (−3.28, 0.49)	−4.30 ± 2.02 (−5.14, −3.47)	0.45 ± 0.86 (0.13, 0.78)	< 0.001	0.742	< 0.001	< 0.001	< 0.001

ANCOVA, analysis of covariance; BMI, body mass index, which is calculated as weight in kilograms divided by height in meters squared; IRI, insulin resistance index, which is calculated as insulin level (mIU/L) × fasting glucose (mmol/L)/22.5.

SI conversions: To convert glucose from mg/dL to mmol/L, multiply by 0.0555; insulin from μIU/mL to pmol/L, multiply by a 6.945.

a *P-value for the omnibus analysis testing for overall differences between the three groups on the continuous variables is based primarily on ANCOVA with baseline levels of the variables as covariates. When the overall omnibus analysis P-value was significant, the pair-wise comparisons were performed*.

At the end point, patients in metformin group had a significant decrease in body weight by 5.79% compared with baseline, while body weight in betahistine group decreased by 1.32% without significance; however, patients in placebo group significantly gained 5.51% of their body weight. Correspondingly, in metformin group, the mean BMI significantly decreased by 1.46, while the mean BMI decreased by 0.33 in betahistine group with no significance, and the mean BMI increased by 1.27 in placebo group significantly.

### Changes in waist circumference

As shown in Table 3, at the end point, the mean waist circumference in metformin group significantly decreased by 1.52 cm, while placebo group had a significant increase of mean waist circumference by 2.29 cm. However, in betahistine group, the mean waist circumference increased with no statistical significance compared with baseline. Both metformin and betahistine were significantly superior to placebo in terms of waist circumference value. However, no significant difference was found between betahistine and metformin (*p* = 0.058).

### Changes in fasting glucose, insulin, and IRI

Over the 12-week period, there was a significant decrease in the values of mean fasting glucose level, insulin level, and IRI in metformin group; however, in betahistine group, no significant change in the value of fasting glucose level, insulin level, or IRI was found compared with baseline, while in placebo group, there was a significant increase in insulin level and IRI but not fasting glucose level (Table 2). At the end point, metformin was superior to both betahistine and placebo in terms of fasting glucose level, insulin level, and IRI, while betahistine was superior to placebo in terms of insulin level and IRI but not fasting glucose level (Table 3).

### Adverse events

There were no significant differences in the frequency and types of adverse events reported among the three groups. There were six serious adverse events that affected more than 5% of the entire sample (Table 4).

**Table 4:** Adverse effects of three groups.

	**No. (%)**			
**Adverse effect**	**Total (*n* = 674)**	**Betahistine (*n* = 13)**	**Metformin (*n* = 25)**	**Placebo (*n* = 29)**	***P*-value**
Nausea	11(16.4)	0(0)	5(20.0)	4(13.8)	0.6
Extrapyramidal Symptoms	17(25.4)	2(15.4)	5(20.0)	8(27.6)
Insomnia and agitation	13(19.4)	1(7.7)	5(20.0)	5(17.2)
Somnolence	7(10.4)	1(7.7)	2(8.0)	2(6.9)
Headache	6(9.0)	2(15.4)	2(8.0)	2(6.9)
Dry mouth	6(9.0)	0(0)	2(8.0)	2(6.9)

*Fisher's exact test among three groups*.

## Discussion

This study was designed to test the comparative efficacy of metformin and betahistine on preventing further weight gain or causing weight decrease in people with schizophrenia or bipolar disorder who had gained more than 10% weight in the first 3 years of treatment with antipsychotics. To our knowledge, this is the first research to compare metformin and betahistine in treating antipsychotic-induced weight gain. After a 12-week trial, we found that betahistine treatment effectively controlled antipsychotic-induced weight gain, while metformin significantly relieved antipsychotic-induced weight gain. Both metformin and betahistine were found to have a significant advantage when compared with placebo.

Current interventions to minimize antipsychotic-induced weight gain and metabolic syndrome include pharmacologic and non-pharmacologic ways (26). Pharmacologic interventions include switching to another antipsychotic, which has less weight gain effect, or adding an adjuvant. However, individuals who switched antipsychotics had significantly shorter times until discontinuation compared with individuals who continued with their baseline medication (27). Therefore, the risk of relapse should be carefully considered before medication switching (28). Meanwhile, non-pharmacological interventions usually consist of lifestyle intervention and cognitive behavior strategies (29). However, there is a significant heterogeneity in non-pharmacological interventions, and the majority of these interventions are associated with poor compliance. So, adding an adjuvant should be a chance to improve antipsychotic-induced weight gain and insulin resistance.

The potential clinical effect of betahistine on reducing antipsychotic-induced weight gain and its mechanism has gained more attention in recent years. The histamine system has played a crucial role in the regulation of energy homeostasis (18, 30, 31). Specifically, H1R antagonism has been recognized as the main mechanism for predicting weight gain induced by second-generation antipsychotics (SGAs) (18, 32, 33). Betahistine, as a H1R and H3R antagonist, can cross the blood-brain barrier, and it acts centrally by enhancing histamine neurotransmission in the hypothalamus (34). A previous animal study has suggested that co-treatment of betahistine could partially reverse olanzapine-induced body weight gain (19) and hypothalamic H1R pathway change (20). The clinical application of betahistine against weight gain has thus been the focus. In a multicenter randomized controlled trial (RCT) study of healthy women, Barak et al. (35) reported that in over 12 weeks of treatment with betahistine, there was a significant weight loss (35). Later, in 2016, their study showed that the coadministration of betahistine and olanzapine mitigated the weight gain induced by olanzapine in healthy women (21). In patients diagnosed with schizophrenia, Poyurovsky et al. (36) held a study for 6 weeks with the coadministration of betahistine and olanzapine, which demonstrated a increase in weight during the initial 2 weeks of the trial with no additional weight gain or minor reduction of body weight for the rest of the trial, and none of the patients gained 7% of the initial body weight (36). Another study by Poyurovsky et al. (37) showed that the reboxetine-betahistine combination produced a significant attenuation of olanzapine-induced weight gain (37), and the weight attenuating effect of this combination was two-fold higher than reboxetine alone (38). Another study carried out on female obese women demonstrated the beneficial effect of betahistine on improving dyslipidemia (39). However, a 1-day administration of betahistine in healthy women showed no difference in energy intake (40). Our research was inconsistent with most of the previous studies and for the first time showed that the treatment of betahistine could mitigate the increased insulin level and IRI induced by antipsychotics.

Metformin mainly increases the function of insulin in the liver and decreases the rate of hepatic glucose production (41). Metformin is regarded as the first line treatment for type 2 diabetes mellitus (42); besides, it was also used in non-diabetics against obesity. In the hypothalamus, metformin increases STAT3 signaling while it decreases NPY and AgRP expression, which suggests that metformin mediated food intake by affecting multiple appetite regulatory pathways (43, 44). Metformin also improves leptin sensitivity, which is an important adipocyte-derived hormone that regulates energy balance (45). Other researchers suggested that metformin could increase the secretion of GLP-1, a satiation signal secreted by the gut (46). In our previous study, it was found that lifestyle intervention and metformin alone and in combination were effective for reversing antipsychotic-induced weight gain, while metformin alone was more effective for inducing weight loss and improving insulin sensitivity than lifestyle intervention alone, and metformin remained effective and safe in attenuating olanzapine-induced weight gain and insulin resistance in drug naïve first episode patients (15). As a follow-up to our initial study, we found that the addition of metformin to antipsychotics was a potential treatment for dyslipidemia in people with schizophrenia (13) and amenorrhea in females with schizophrenia (14, 47). Multiple compounds have been investigated as add-on medications to cause weight loss, and metformin has the best evidence (26). However, in 2018, a meta-analysis that included six RCTs found that combining metformin and lifestyle interventions shows significant reduction in weight and BMI compared with metformin alone (48). Three metformin meta-analyses confirmed the significant effect of metformin in reducing BMI and improving insulin sensitivity (49–51). Our findings were consistent with most of these studies and meta-analyses.

In addition, our study showed that metformin significantly decreased body weight, BMI, fasting glucose level, insulin level, and IRI but not waist circumference when compared with betahistine. These findings suggested that the treatment with metformin could be more efficacious than betahistine in preventing and reversing the weight gain induced by antipsychotic agents in people with schizophrenia or bipolar disorder, while both treatments were found to have a significant advantage over placebo. Few studies had compared the effect of betahistine and metformin before. According to our study, although betahistine group failed to decrease the body weight significantly, it prevented further weight gain with a decreasing tendency. Therefore, we suggest metformin as the first consideration for antipsychotic-induced weight gain while betahistine as an alternative if metformin was not tolerated or adhered.

This study has some limitations. First, the data were collected from two independent studies, thus, the sample error was inevitable and STUDY 2 was not a randomized placebo controlled clinical trial. Second, this study was based on schizophrenia or bipolar disorder participants with four different antipsychotics: clozapine, olanzapine, risperidone, or quetiapine. Previous studies suggested that the type of antipsychotics affects the plasma adiponectin level and also affects body weight significantly (52, 53). However, we were unable to assess this effect by the type of antipsychotics because of the small sample size in our study. Third, we failed to test leptin level though it has been proven to play an important role in weight gain (54). Finally, the participants were followed up for 12 weeks only, so we still cannot predict the long-term effects of metformin and betahistine. Further research including the well-designed RCT test to testify the findings or genetic variations, which might provide some explanation on individualized treatment response, should be carried out.

In conclusion, despite these limitations, this study has clearly shown that metformin could be more efficacious than betahistine in increasing insulin sensitivity and reversing the weight gain induced by antipsychotics with 12 weeks of treatment, while both could significantly improve the body weight and insulin sensitivity induced by antipsychotics. We suggest metformin as the first consideration for the treatment while betahistine as an alternative if not tolerated or adhered.

## Author contributions

RW and ZJ designed and conducted the research. DK analyzed and interpreted data and drafted the article. RW, ZJ, and RL provided critical revision of the article, while GH, TS, ZJ, RL, LL, MS, YY, YW, XW, and YL collected and analyzed the data. XH analyzed the data and drafted the data. RW are responsible for the final approval of the version to be published.

### Conflict of interest statement

The authors declare that the research was conducted in the absence of any commercial or financial relationships that could be construed as a potential conflict of interest.
